# PB.47: Mammography in symptomatic women aged 35 to 39 years

**DOI:** 10.1186/bcr3547

**Published:** 2013-11-08

**Authors:** VW Chan, MG Bell, AL Brown

**Affiliations:** 1 Royal Berkshire NHS Foundation Trust, Reading, UK

## Introduction

Until recently, in our institution, all women over the age of 35 years referred to the symptomatic breast clinic for triple assessment undergo mammography. Best practice diagnostic guidelines for patients presenting with breast symptoms (Department of Health November 2010) recommend that mammograms should routinely be performed in women aged over 40 years. The aim of this study was to assess the impact that changing our policy would have on breast cancer detection in women aged 35 to 39 years.

## Methods

Our Breast Cancer Database identified all women aged 35 to 39 years diagnosed with breast cancer between April 2003 and December 2012. A retrospective review of breast imaging and reports on PACS was carried out to determine whether ultrasound alone would have detected the malignancy.

## Results

106 cancers were diagnosed in 103 women aged 35-39 years. In 103 cases, ultrasound scores using the RCR Breast Group Classification ranged from U3 (indeterminate/probably benign) to U5 (highly suspicious of malignancy). Two patients presented with nipple discharge. One of these patients had mammographic microcalcification due to extensive DCIS with small separate foci of invasive cancer with normal ultrasound examination.

## Conclusion

In our institution, one case of extensive DCIS with small separate foci of invasive cancer out of a total of 106 breast cancers would have been missed on breast imaging if routine mammography had not been performed in women aged 35-39 years.

